# Fast retrospectively triggered local pulse-wave velocity measurements in mice with CMR-microscopy using a radial trajectory

**DOI:** 10.1186/1532-429X-15-88

**Published:** 2013-10-01

**Authors:** Patrick Winter, Thomas Kampf, Xavier Helluy, Fabian T Gutjahr, Cord B Meyer, Rommel, Wolfgang R Bauer, Peter M Jakob, Volker Herold

**Affiliations:** 1 Julius-Maximilians-Universität Würzburg, Lehrstuhl für Experimentelle Physik 5, Am Hubland, 97074, Würzburg, Germany; 2 Julius-Maximilians-Universität Würzburg, Medizinische Universitätsklinik, Josef-Schneider Straße 4, 97080, Würzburg, Germany

**Keywords:** Pulse-wave velocity, Mouse, Self-gating, Phase-contrast CMR, Non-triggered, Retrospective, Radial, Aorta

## Abstract

**Background:**

The aortic pulse-wave velocity (PWV) is an important indicator of cardiovascular risk. In recent studies MRI methods have been developed to measure this parameter noninvasively in mice. Present techniques require additional hardware for cardiac and respiratory gating. In this work a robust self-gated measurement of the local PWV in mice without the need of triggering probes is proposed.

**Methods:**

The local PWV of 6-months-old wild-type C57BL/6J mice (n=6) was measured in the abdominal aorta with a retrospectively triggered radial Phase Contrast (PC) MR sequence using the flow-area (QA) method. A navigator signal was extracted from the CMR data of highly asymmetric radial projections with short repetition time (TR=3 ms) and post-processed with high-pass and low-pass filters for retrospective cardiac and respiratory gating. The self-gating signal was used for a reconstruction of high-resolution Cine frames of the aortic motion. To assess the local PWV the volume flow *Q* and the cross-sectional area *A* of the aorta were determined. The results were compared with the values measured with a triggered Cartesian and an undersampled triggered radial PC-Cine sequence.

**Results:**

In all examined animals a self-gating signal could be extracted and used for retrospective breath-gating and PC-Cine reconstruction. With the non-triggered measurement PWV values of 2.3±0.2 m/s were determined. These values are in agreement with those measured with the triggered Cartesian (2.4±0.2 m/s) and the triggered radial (2.3±0.2 m/s) measurement. Due to the strong robustness of the radial trajectory against undersampling an acceleration of more than two relative to the prospectively triggered Cartesian sampling could be achieved with the retrospective method.

**Conclusion:**

With the radial flow-encoding sequence the extraction of a self-gating signal is feasible. The retrospective method enables a robust and fast measurement of the local PWV without the need of additional trigger hardware.

## Background

Cardiovascular Disease (CVD) is still one of the main causes of death in industrial nations. One important indicator of cardiovascular risk is the aortic pulse-wave velocity (PWV) as a measure of arterial stiffness [1,2].

In the past years genetically modified mice have turned out to be of great relevance as models for the research of this predictor for the formation of CVD. As a non-invasive method high-field CMR has gained great importance for the measurement of morphological and functional parameters of the cardiovascular system in small animals [3]. A triggered Cartesian PC-Cine sequence was proposed which uses the flow-area (QA) method to determine the local PWV [4]. In this method high spatial and temporal resolutions were achieved by the acquisition of multiple interleaved Cine experiments. With the development of more capable hardware and faster reconstruction algorithms non-Cartesian sequences were developed to accelerate the CMR experiment and improve CMR robustness against flow artifacts. Zhao et al. improved the temporal resolution and acquisition time for a measurement of the regional PWV by measuring flow in multiple slices with a triggered undersampled and asymmetric radial Phase Contrast MR sequence [5].

A drawback of prospectively triggered methods is the necessity of external probes, which monitor cardiac and respiratory motion. The most common method to trigger the CMR acquisition is the use of Electrocardiograms (ECG). However, rapid switching of the imaging gradients can cause interferences with the ECG probes, which can strongly corrupt the trigger signal [6]. In addition, in high field CMR, the small RF coil dimensions can make the attachment of triggering probes difficult. Therefore experience with animal handling is required to ensure a stable trigger signal. The use of the CMR signal itself offers the possibility of a wireless trigger signal, which does not require external probes and is therefore easier to handle. Since the peak value of the echo signal corresponds to the sum of the transverse magnetization across the whole field of view (FOV), moving and changing voxels cause fluctuations of the peak signal intensity corresponding to the motion [7]. The signal variations can be assigned to respiratory and cardiac motion within the excited slice. With the application of fast repetitive radial read-outs a navigator signal can be extracted which can be used for retrospective triggering. This technique was used for the generation of self-gated cardiac Cines in mice without additional hardware [8].

The goal of our work was to design a fast self-gated PC-Cine method for PWV measurements in the aortae of mice, which is independent of additional trigger hardware and is applicable to high field CMR, where susceptibility disturbances are significantly increased. Since a stable trigger signal is required for the determination of this parameter, the self-gated method promises to improve the robustness of the measurement. However, the challenge for flow CMR in the aorta without triggering probes is that the slice of interest is often located remotely from the beating heart and therefore might contain only a small portion of changing voxels, which are able to contribute to a self-gating signal. Additionally, high temporal and spatial resolutions as well as small echo times in order to reduce image artifacts are necessary, which imposes high requirements to the flow compensated spatial encoding gradients. To ensure high-resolution measurements and reduced sensitivity to susceptibility variations, a highly asymmetric radial pulse sequence was developed to enable the acquisition of a self-gating signal without prolonging the read-out.

## Methods

### CMR

#### Pulse sequences

**Figure 1:**
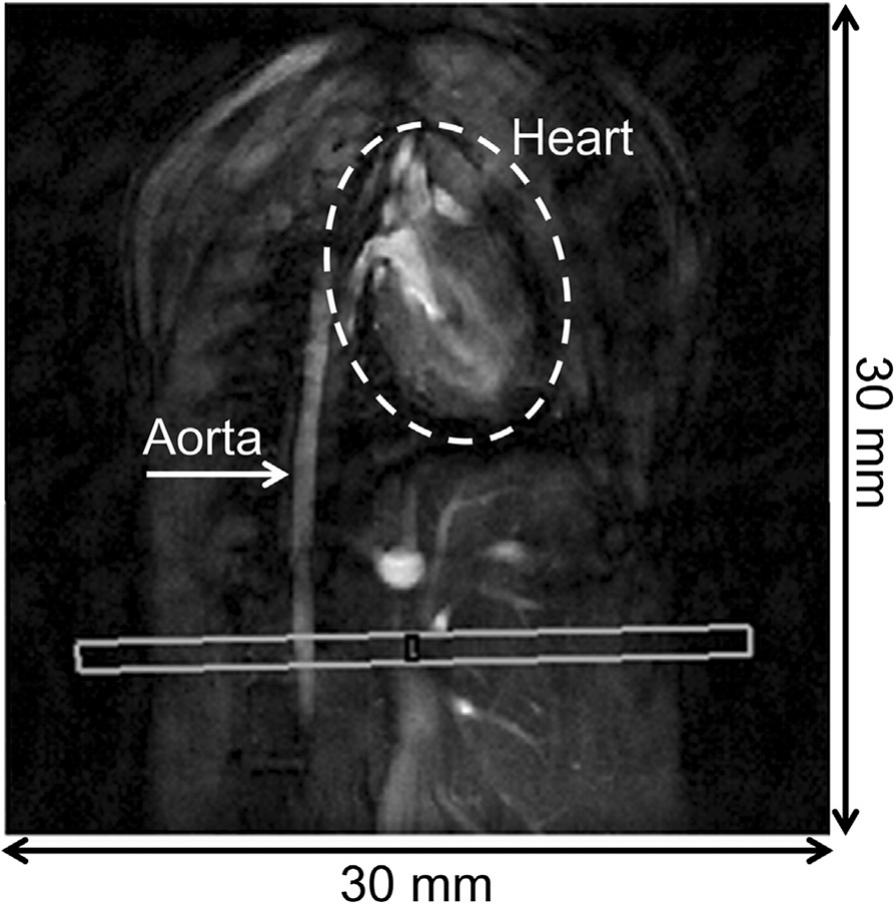
Slice positioning for the local PVW measurements in the abdominal aorta.

**Figure 2:**
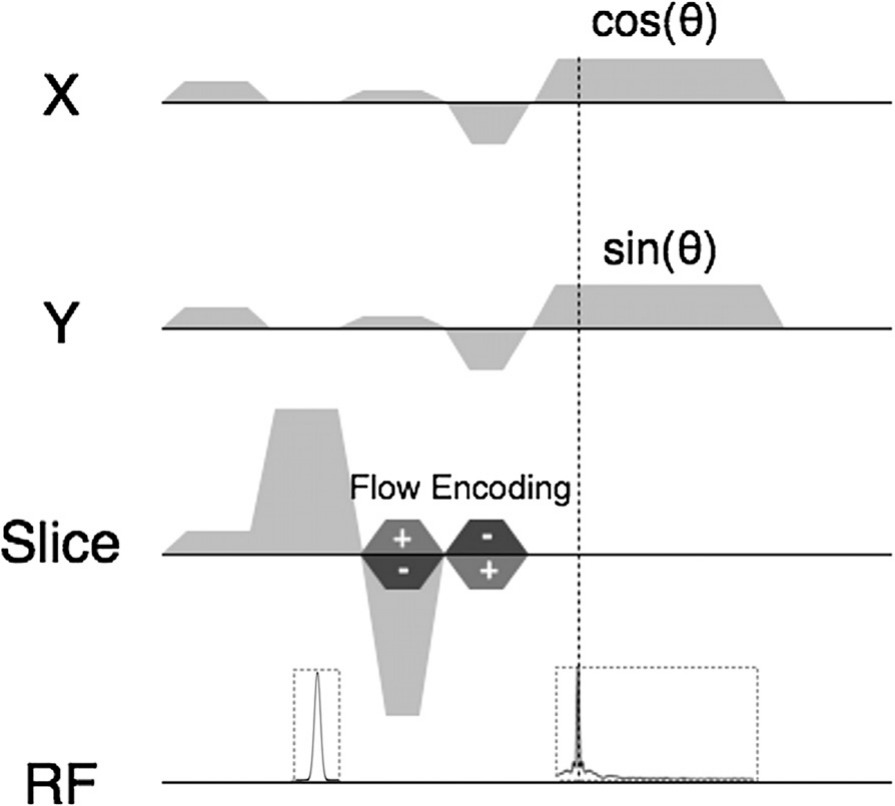
**Pulse-sequence for the retrospective measurement of blood flow and the cross-sectional areas.** The measurement is motion-compensated in all three encoding directions. Flow measurements are done with a bipolar gradient in slice direction, which is added to the slice-selection gradient. After gradient spoiling and slice selection tripolar gradients are applied in the logical X- and Y-direction with *G*_*x*_=*G*·cos(*θ*) and *G*_*y*_=*G*·sin(*θ*), where *θ* is the projection angle and *G* the maximum strength of the read-out gradient. The gradient echo is acquired at a position of 10% of the read-out duration.

All measurements are carried out at a 17.6 T small animal system (Bruker Avance 750 WB, Bruker BioSpin MRI GmbH, Rheinstetten, Germany) with a 1 T/m gradient system and a home-built transverse electromagnetic (TEM) resonator with an inner diameter of 25 mm. The imaging protocol started with 2D-FLASH experiments to localize the descending aorta. The local PWV was measured in a slice perpendicular to the vessel (see Figure 1). Based on the Cartesian Phase Contrast sequence proposed in [9] a motion-compensated radial PC-Cine-FLASH sequence was designed for through-plane velocity encoding (see Figure 2). The scan protocol consists of three sequentially applied velocity-encoding steps with varying first gradient moments (M_1_=0 s/m and M_1_=±0.3 s/m), corresponding to a maximum encoding velocity (VENC) of 1.7 m/s. The radial sequence enables either a triggered flow-encoding mode based on the interleaved acquisition scheme of [4] or retrospective flow measurements without triggering.

To assess the quality of the retrospectively triggered PC-Cine measurement, prospectively triggered Cartesian and radial PC-Cine experiments were conducted for comparison. The Cartesian measurement was acquired using a 160×160 matrix and a field of view of 24×24 mm^2^. The radial data was reconstructed on the same 160×160 grid with the same field of view and spatial resolution as with the Cartesian experiment. All images were zero-filled to a matrix size of 256×256 for the following evaluation. The slice thickness was 1 mm in all experiments. Further relevant parameters of all three measurement types are listed in Table 1.

**Table 1:** Scan parameters for the three flow-encoding sequences

**Parameters**	**Cartesian**	**Radial triggered**	**Retrospective**
**TR [ms]**	5	3	3
**TE [ms]**	1.7	1.2	1.2
**Sampling bandwidth [kHz]**	75.8	75.8	75.8
**Flip angle*[°]***	30-40	30-40	30-40
**Echo position**	25	10	10
**[% of the read-out]**			
**Number of read-out points**	160	90	90
**Number of projections**	160	160	16000*
**Number of Cine segments**	5	3	-
**Frames per Cine Segment**	8	14	-
**Number of velocity-encoding steps**	3	3	3
**VENC [m/s]**	1.7	1.7	1.7
**Spatial resolution*[μ*m**^**2**^***]***	150×150**	150×150^*‡*^	150×150^*‡*^
**Field of view [mm**^**2**^**]**	24×24	24×24	24×24
**Slice thickness [mm]**	1	1	1
**Measurement time [minutes]**	6.7±1.1	4.3±0.6	2.9

^*^Including 500 dummy scans to establish the steady state. **Zero-filled to a 256 × 256 matrix. *‡*Reconstructed on a 160 × 160 grid and zero-filled to a 256 × 256 matrix.

#### Retrospectively triggered measurement

For the retrospective triggering mode a train of n_Proj_=16000 radial projections per velocity-encoding step was acquired without cardiac and respiratory gating with a short repetition time (TR=3 ms).

To reduce the echo time and therefore the strong influence of B_0_ inhomogeneities at 17.6T, which are especially significant in vessels close to the lung, the echo was set to 10% of the read-out duration by decreasing the areas of the read dephase gradients (see Figure 2). A waiting period of 15 s was applied between each velocity-encoding step to reduce the effect of RF coil heating and SAR due to the fast application of RF pulses [10]. The total scan time including the waiting times was 3 minutes.

#### Prospectively triggered measurement

All prospectively triggered measurements were done with a pressure sensitive balloon to measure the thoracic motion during breathing and the heartbeats. The trigger signal was post-processed with an amplification unit (Rapid Biomedical, Rimpar, Germany). With the triggered Cartesian measurement an effective temporal resolution of 1 ms was achieved by the acquisition of 5 interleaved Cines with 8 movie frames each, covering 40 ms of the heart cycle around the systole. The total scan time depends on breathing and the heart rate and was 6-8 minutes in this study. For the prospectively triggered radial measurement three interleaved Cines with 14 movie frames each were acquired to obtain the same temporal resolution as for the Cartesian measurement, covering 42 ms of the heart cycle. For each image 160 equally distributed spokes were acquired, therefore the undersampling factor was af ≈3.12, respective to the Nyquist criterion. The total scan time was 3-5 minutes.

#### Gradient delay correction

As a non-Cartesian imaging method the quality of the radial measurement is compromised by trajectory errors caused by hardware imperfections such as delays of the spatial encoding gradients. To improve image quality, the gradient switching times were determined priorly with a homogenous CuSO_4_ doped water phantom using two different methods as proposed in [11] and [12], respectively. Measurements using either technique yielded gradient delay values of about *t*_*x*_=8 *μ*s for the X-, *t*_*y*_=10 *μ*s for the Y- and *t*_*z*_=8 *μ*s for the Z-gradient. The measured parameters were afterwards used for a correction of the gradient areas by adjusting the amplitudes of the read dephase gradients, as described in [11].

### Trigger signal processing

For the retrospective reconstruction several steps were performed to obtain the trigger signal:

#### Modified golden angle based radial read-out

In mice anesthetized with isoflurane the heart typically contracts with a repetition rate of 300-600 beats per minute, which is usually too fast for golden-ratio based real time Cine applications [13]. Therefore, to achieve sufficient SNR and temporal resolution, the aortic motion is monitored over several seconds. For the reconstruction of a Cine frame, the projections acquired during many heart beats were afterwards sorted by their phase in the cardiac cycle and used to fill the k-space. Since a standard golden-ratio based angular distribution might result into an undesired clustering of the retrospectively selected projections, a modified golden angle based read-out was precalculated.

**Figure 3:**
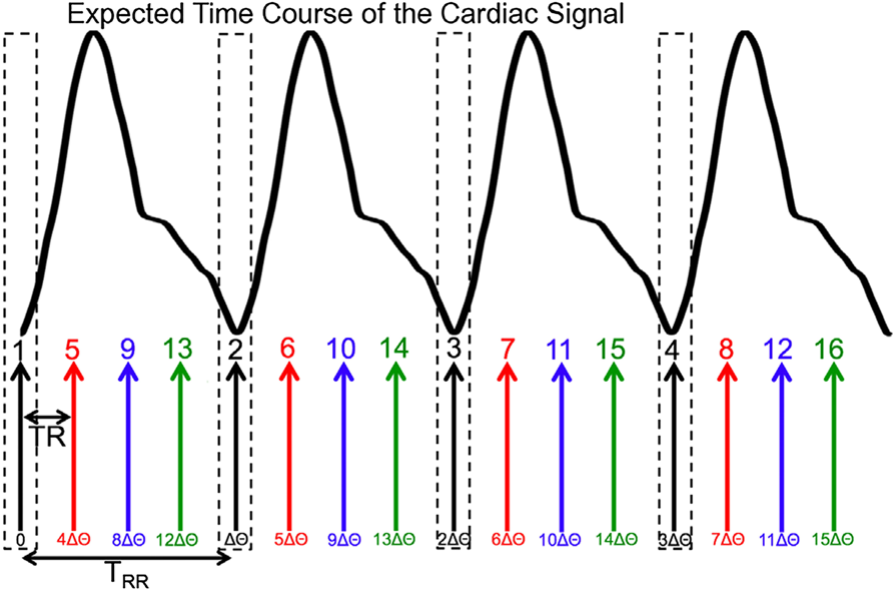
**Precalculation of a golden-ratio based angular distribution to achieve a more uniform coverage of k-space with the projections selected for Cine reconstruction.** Above: Expected time course of the cardiac signal, estimated from the average interval T_RR_. Below: Using *θ*_0_=0° as initial value, a list of projection angles was calculated by consecutively adding of the golden angle (*Δθ*≈111.246°). Subsequently, the projections of the calculated angle list were sorted into N≈T_RR_/TR interleaved sublists (demonstrated for N=4 sublists). Each sublist consists of K≈n_Proj_/N golden-ratio-distributed projections (shown for K=4). In case of a constant signal each projection of one sublist belongs to the same heart cycle phase, as shown for the systolic phase and the black projections.

Using *θ*_0_=0° as initial value, an angle list was calculated by consecutively adding the golden angle *Δθ*≈111.246° [13]. Assuming that the variations from the average interval T_RR_ between two heart beats are small, the angles were afterwards sorted into N ≈T_RR_/TR interleaved sublists using the sorting:

(1) $$\theta_{n}(k\cdot T_{\text{RR}})=\theta_{n,0}+k\cdot \Delta \theta ,$$

where *n*=1,…,N is the number index of the sublists, $k=1,\ldots ,\text{K}\approx \frac{n_{\text{Proj}}}{N}$ the index of the projections per sublist and *θ*_*n*,0_=*θ*_n-1_(K·T_RR_) the last angle value of the previous sublist. Figure 3 and an additional animation in the supplement show the used sorting in more detail (Additional file 1). The idea of this sorting of projection angles is that in case of an almost constant heart rate a large amount of retrospectively selected projections for one time frame would belong to the same sublist and would therefore be golden-ratio distributed. An estimate of the average interval T_RR_ was assessed with the pressure sensitive balloon signal, which was observed with the trigger unit to monitor cardiac and respiratory motion of the animal. Depending on the heart rate, the measured projections consist of 33-38 sublists with 420-475 golden angle-distributed projections per sublist.

#### Signal processing

For retrospective triggering the k-space center of the radial projections was taken. The real part, the imaginary part and the magnitude values were investigated to find the trigger signal with the strongest modulation due to blood flow. The first 1.5 s (i.e., 500 projections) of the data were excluded from data processing since during this time the signal shows a strong transient to the steady state.

**Figure 4:**
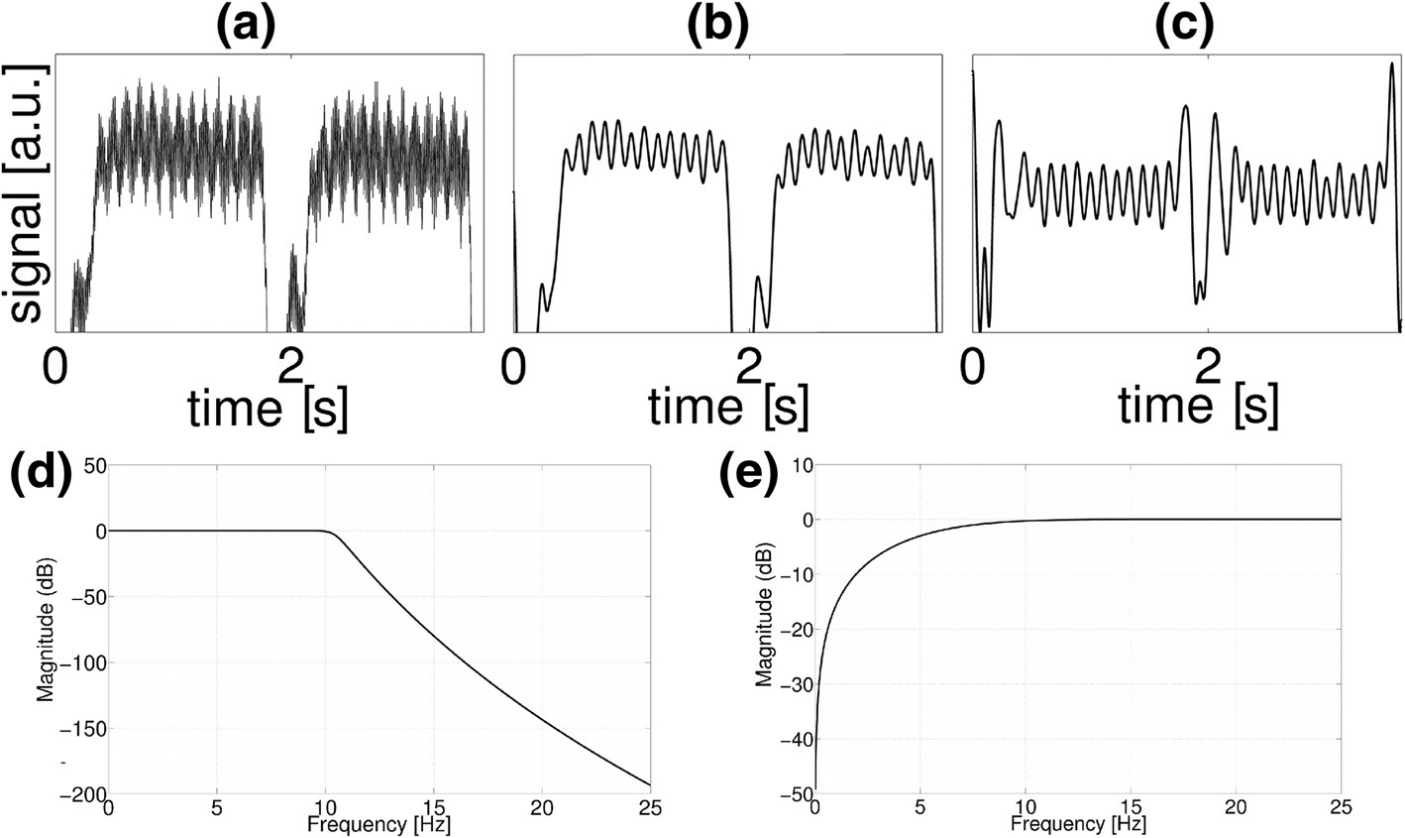
**Filtering of the radial self-gating signal.(a)**: The raw signal is disturbed by higher harmonic oscillations. **(b)**: A Butterworth low-pass filter is applied to suppress high frequency interferences. **(c)**: Low frequency oscillations caused by respiratory motion are reduced with a gaussian-shaped window function. **(d)**: Magnitude response of the low-pass filter. Used pass band frequency: 10–13 Hz. Stop band frequency: 15-20 Hz. **(e)**: Magnitude response of the Gaussian window function (*w*(*f*)=1-exp(-(*f*/*σ*)^2^)). The used window width was *σ*=4-8 Hz.

Since the original signal is often compromised by high harmonic disturbances (Figure 4a), a Butterworth low-pass filter [14] was applied to suppress high-frequency interferences (F4b). Afterwards a Gaussian-shaped window function was used to reduce low-frequency modulations caused by respiratory motion (F4c). F4d and e show the magnitude responses of the used frequency filters. For the Butterworth filter a pass band frequency of 10-13 Hz and a stop band frequency of 15-20 Hz were found to provide the best filtering results. The high-pass filter has the shape *w*(*f*)=1-exp(-(*f*/*σ*)^2^), with a window width of *σ*=4-8 Hz.

#### K-space selection

**Figure 5:**
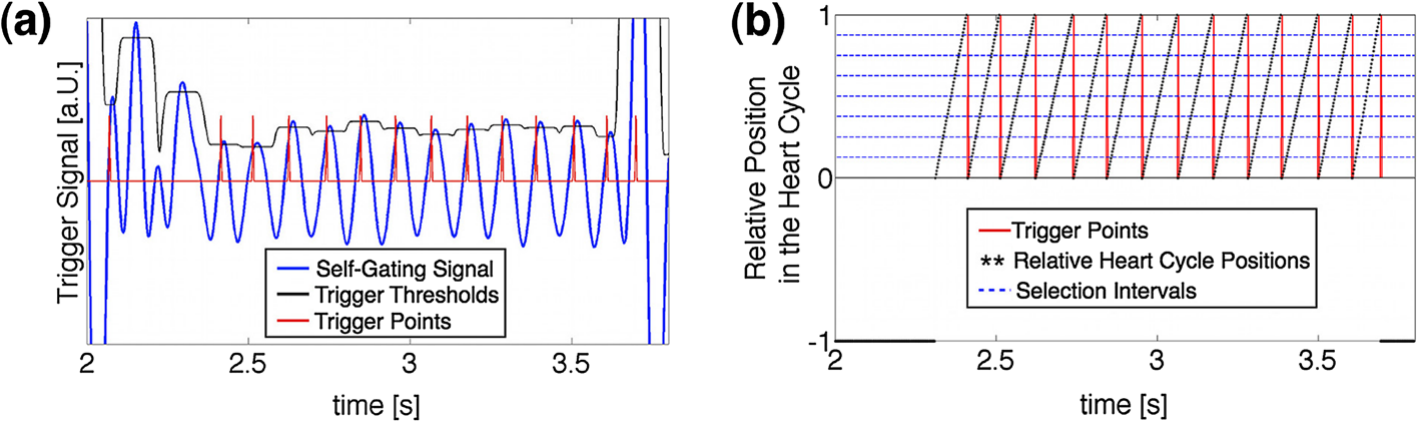
**Determination of the trigger points.(a)** Definition of trigger thresholds (black). As threshold value 90% of the local maxima of the cardiac signal (blue) is taken. As trigger points (red) always the first value above the threshold is chosen. **(b)** After definition of the trigger points (red) each read-out is assigned to a relative position in the heart cycle between 0 and 1 (black). Data during respiratory motion is gated out (relative position =-1). For projection selection the relative positions in the heart cycle were divided into 100 intervals (blue, displayed for 8 intervals).

For the assignment of the acquired projections to a corresponding heart cycle phase, the trigger signal extracted from the radial k-space data, which was acquired with a sampling rate of TR=3 ms, was interpolated to 1/10 of TR using a linear interpolation. To select a particular phase within the heart cycle, trigger points were determined with thresholds (threshold value: 90% of the local maximum of the interpolated self-gating signal). The first value above the threshold was chosen as the trigger point (Figure 5a). After determination of the trigger positions each radial projection was assigned to a relative position in the heart cycle between 0 and 1 (see Figure 5b). Data points during respiratory motion were gated out (relative position = -1). For the reconstruction of relative time frames, the relative heart cycle positions were divided into 100 equidistant selection intervals (Figure 5b). Projections located within a temporal distance of ±0.5 ms around the corresponding heart cycle phase were assigned to each selection interval. Using this sliding window selection, an average number of about 120 projections per image were assigned to 100 time frames of the aortic motion covering the whole heart cycle.

### Reconstruction

All radial reconstructions were done in MATLAB (The MathWorks, Inc., Natick, MA) with a Non Uniform Fast Fourier Transform (NUFFT, [15]) using the software toolbox provided by Fessler et al. [16]. The complex k-space data was re-gridded with a Kaiser-Bessel window [17]. The density compensation function was calculated using the iterative algorithm described by Pipe et al. [18].

Due to imperfect shimming a retrospective correction of B_0_ offsets and linear inhomogeneities was applied for reconstruction. Therefore an off-resonance map was determined through the acquisition of two triggered gradient-echo images *ϱ*_1_ and *ϱ*_2_ with a relative echo time difference of *Δ*TE=0.5 ms, as described in [19]. With the phase of the conjugate complex product, $\Delta \Phi (\vec{r})=\text{angle}(\varrho_{1}^{*}\cdot \varrho_{2})$, the off-resonance frequencies can be calculated using $\Delta \omega_{0}(\vec{r})=\frac{\Delta \Phi (\vec{r})}{\Delta \text{TE}}$. A phase unwrapping was applied, when necessary [20]. Using this a priori information the off-resonance corrected image $\hat{\varrho }(\vec{r})$ can be estimated with the conjugated phase reconstruction proposed in ( [21], Eq. 7):

(2) $$\hat{\varrho }(\vec{r})=\underset{j}{\sum }s(t_{j})e^{\mathrm{i\Delta}\omega_{0}(\vec{r})t_{j}}C(\;j,\vec{r}).$$

Hereby $\underset{j}{\sum }$ denotes a summation over the measured k-space points, *s*(*t*_*j*_) the measured CMR signal and *t*_*j*_ the discrete sampling times of the measurement. $C(\;j,\vec{r})=e^{i\vec{k}(t_{j})\vec{r}}w_{j}$ is a complex weighting matrix which contains the information about the k-space trajectory $\vec{k}(t_{j})$ and the real-valued sampling densities w_*j*_ calculated with the Pipe algorithm [18]. Since an exact execution of the conjugated phase reconstruction has high computational requirements due to the large dimensions of the trajectory and the off-resonance term [21], Equation (2) is solved numerically using the NUFFT and an approximation of the exponential term $e^{\mathrm{i\Delta}\omega_{0}(\vec{r})t_{j}}$, which is described in detail in [22,23].

### PWV calculation

For the calculation of the local pulse-wave velocity the flow-area (QA) method proposed by [24] was used. Assuming that a flow pulse through the aorta during the early systolic wave is reflection-free and unidirectional [24], the PWV can be determined using the approximation:

(3) $$\text{PWV}=\frac{\text{dQ}}{\text{dA}},$$

where *dQ* is the change of the volume flow during the early systole and *dA* is the change of the cross-sectional area of the vessel. For a segmentation of the aorta all images were zero-filled to a matrix size of 256×256. The cross-sectional area *A(t)* and the volume flow *Q(t)* were determined using AMIRA (Mercury Computer Systems, Chelmsford, USA) for a segmentation of the vessel for each time frame. All segmentations were done 4 times by the same observer to reduce the standard error. The velocity information was calculated with a linear fit to the phase data of each pixel within the aorta.

For a PWV determination the early upstroke of the measured *Q(A)*-curve was fitted with a linear function. Since the *Q* and *A* data estimated from the reconstructed Cine images is superimposed with high frequency random noise, both curves were smoothed using a moving average filter [25] in the temporal domain, as mentioned in [4].

### Animal handling

A group of 6 C57BL/6J mice (Charles River Laboratories) aged 6 months was imaged in vivo. Anesthetization was done with isoflurane (1.5-2% volume 0_2_ (2L/min)). The temperature was kept constant at 35°C using the gradient cooling unit. All mice were measured vertically (head up). All experimental procedures were in accordance with institutional and internationally recognized guidelines and were approved by the Regierung von Unterfranken (Government of Lower Franconia, Würzburg, Germany) to comply with German animal protection law. The reference number of the permit of the animal experiments is 55.2-2531.01-14/10.

## Results

**Figure 6:**
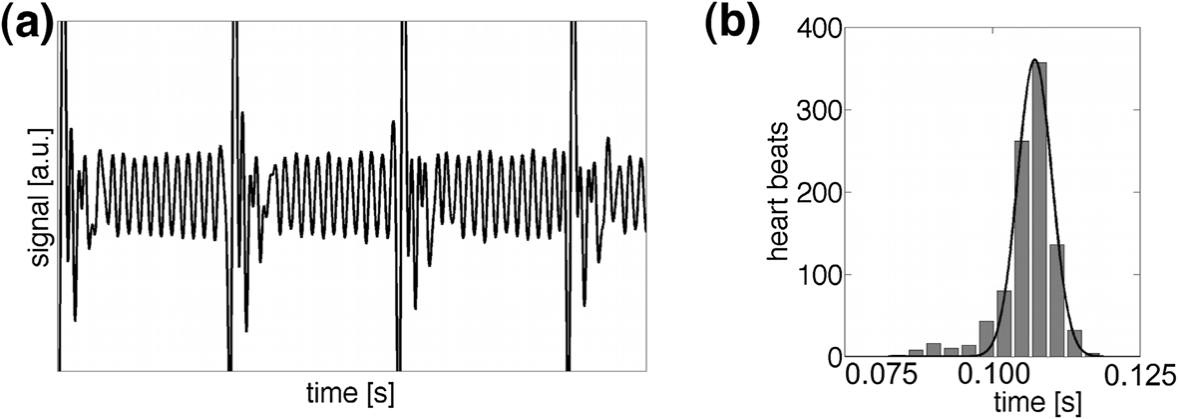
**Measured self-gating signal. (a)** Representative self-gating signal obtained in a C57BL/6J mouse (only motion compensated measurement shown). **(b)** Histogram of the trigger point distances. The navigator signal of all three velocity-encoding steps was taken (3×48 s measurement time). A large narrow peak is visible that can be assigned to the blood flow.

**Figure 7:**
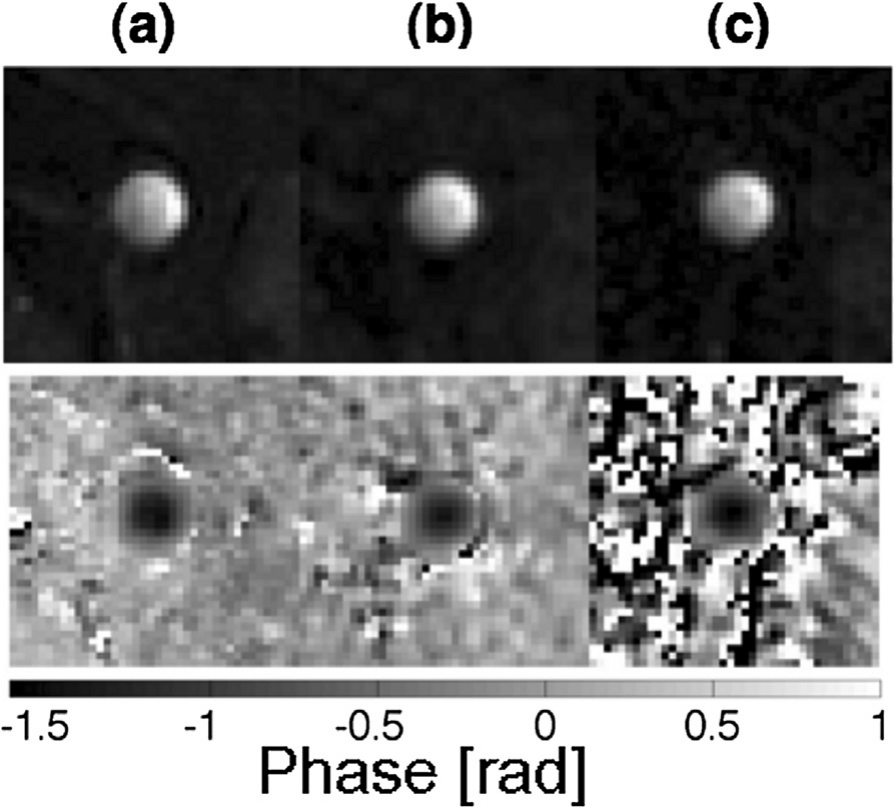
**Images of the abdominal aorta. (a)**-**(c)** above: Representative magnitude images of the motion compensated measurement for the time frame with the maximum velocity, acquired with a triggered Cartesian sampling **(a)**, a triggered radial sampling **(b)** and the retrospective radial measurement **(c)**. The spatial resolution of all images was 150×150 *μ*m^2^, interpolated to 94×94 *μ*m^2^ using zero-filling. **(a)**-**(c)** below: Velocity-encoded PC MR phase images belonging to the same slice and time frame.

**Figure 8:**
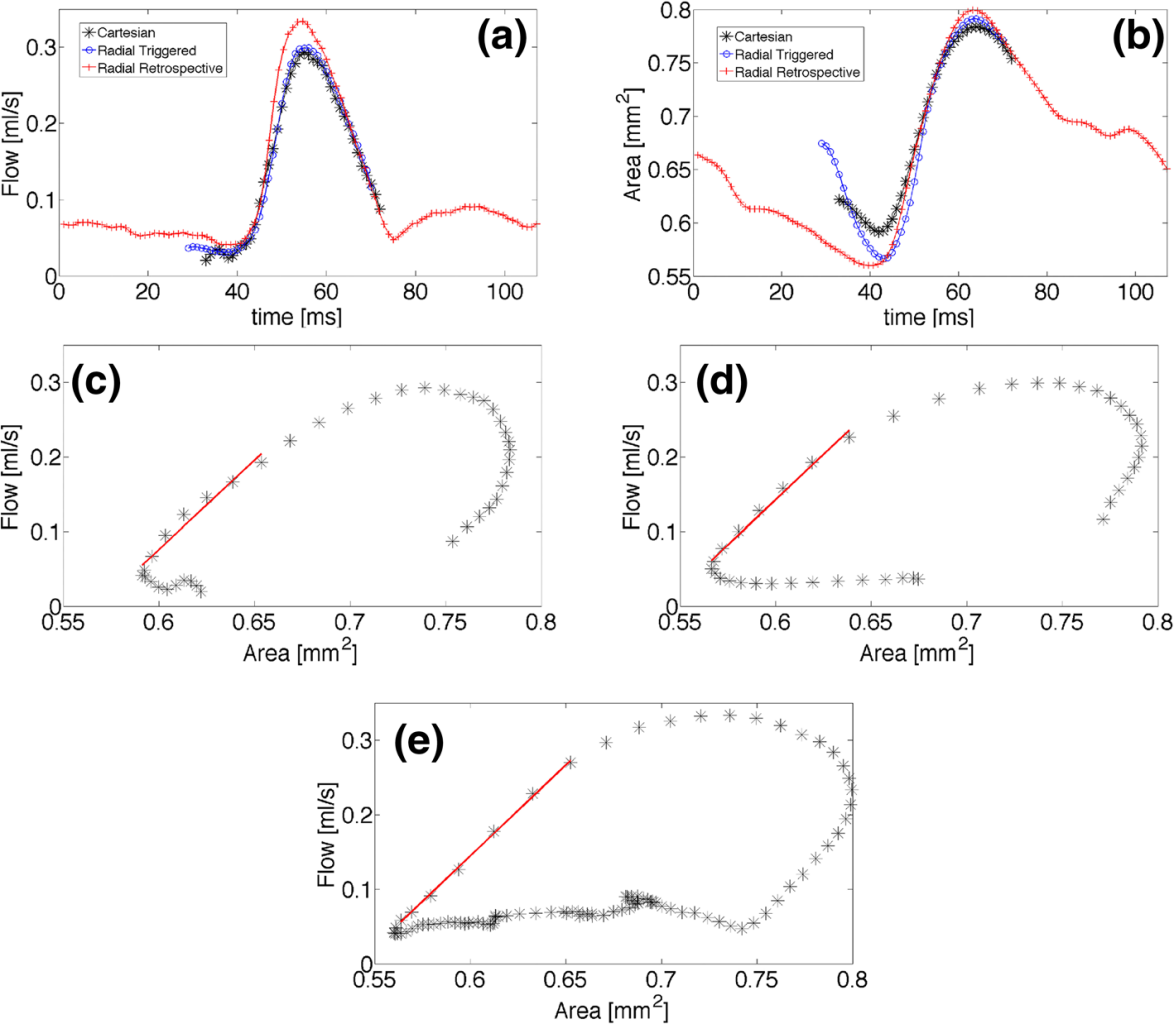
**Results of the QA measurements.** Representative results of a flow-area measurement in the abdominal aorta of a C57BL/6J mouse (age 6 months). Above: Volume flow *Q(t)***(a)** and corresponding cross-sectional area *A(t)***(b)** (overlay of the triggered Cartesian, the triggered radial and the retrospective radial measurement). Below: *Q-A* curves to determine the local pulse-wave velocity. **(c)** With the triggered Cartesian measurement. **(d)** With the triggered radial measurement. **(e)** With the retrospective radial measurement.

Figure 6a displays a representative section of the self-gating signal of a motion compensated measurement (M_1_=0 s/m). The signal modulations due to breathing and the blood flow through the aorta are clearly visible. Figure 6b shows a histogram of the time intervals between each found trigger point, which was calculated from the navigator signals of all three velocity-encoding steps. The average interval between two trigger peaks was 107.2±4.1 ms (mean value and variation estimated with a Gaussian fit). The distribution of the trigger point distances is in range of natural variations of the heart rate. Through the reconstruction of 100 movie frames of the aortic motion an average frame rate of 933±36 frames/s was achieved. In Figure 7 representative flow compensated magnitude images of the aorta during the systolic heart phase are shown for the Cartesian, the triggered radial and the retrospective radial measurement (Figure 7a to c upper row). The scan time was 7 minutes for the Cartesian measurement and 4 minutes for the triggered radial measurement. The retrospective measurement was done in only 3 minutes. For the non-triggered reconstruction an average number of 120 spokes could be used, which corresponds to an undersampling factor of more than 4 respective to the Nyquist criterion. The bottom images of Figure 7a-c show phase maps corresponding to the same measurement. In the phase map of the retrospectively triggered measurement streaking artifacts are visible, however, the aortic flow is only slightly affected by these. The Figures 8a and b display an overlay of the flow (*Q*) and the area (*A*) waveform for the triggered and retrospective measurement. The area curves of the three measurements are slightly shifted among each other due to uncertainties of the aorta segmentation. Also smaller values of the peak volume flow are noticeable with the prospectively triggered measurements, which might be the result of instabilities of the balloon signal. However, for a determination of the PWV only relative changes of the area- and flow curves are important [4]. All three flow-area plots show consistency with the hypothesized linear behavior during the early systole (Figure 8c-e). For a determination of the pulse-wave velocity only the first data points after the beginning of the upstroke are relevant. With each the triggered Cartesian and the triggered radial measurement 8 data points (8 ms) of the early upstroke of the systolic-flow pulse could be used for a linear fit. The frame rate of the retrospective measurement was high enough to take 7 data points (≈7.5 ms) for the fit. Table 2 shows the averaged results for the calculated parameters of all 6 monitored animals. All three methods have sufficient temporal resolution to sample the upstroke of the systolic volume flow with 5-8 data points, respectively. The average PWV determined with the Cartesian measurement was 2.4±0.2 m/s and for the prospectively triggered radial measurement 2.3±0.2 m/s. The PWV measured with the retrospectively triggered method was 2.3±0.2 m/s, which is in good agreement with the results of the prospectively triggered measurements. 

## Discussion

### PWV values

This work shows that a self-gated measurement of the local pulse-wave velocity in the aorta is feasible. The average PWV values of the retrospectively triggered measurement are in good agreement with the triggered Cartesian and radial measurements. In [4] PWV values of 2.6±0.2 m/s are published for 8-months-old WT-mice using the prospectively triggered Cartesian PC-Cine measurement, which is in the same range as the data presented here. Zhao et al. [5] published values of 3.54±0.97 m/s for the PWV of 9-months-old WT-mice. In ultrasound measurements PWV values of 2.86±0.14 m/s are found for 8-month old B6D2F1 mice [26].

**Table 2:** Average results of the local PWV measurements in the abdominal aorta, determined with 6 animals

**Parameters**	**Results**
**Weight (g)**	24.9±3.1
**Peak CSA [ mm**^**2**^]	
Cartesian Measurement	0.75±0.05
Radial Triggered Measurement	0.75±0.04
Retrospective Measurement	0.73±0.04
**Peak volume flow [ cm**^**3**^/s]	
Cartesian Measurement	0.27±0.04
Radial Triggered Measurement	0.28±0.03
Retrospective Measurement	0.28±0.04
**PWV [m/s]**	
Cartesian Measurement	2.4±0.2
Radial Triggered Measurement	2.3±0.2
Retrospective Measurement	2.3±0.2

Results are presented as mean ± SE. PWV: Pulse-wave velocity. CSA: Cross-sectional-area.

#### Trigger quality

In contrast to measurements close to the beating heart only view moving voxels can be found in a slice in the abdominal region. Therefore the waveform of the navigator signal is less distinctive then in a cardiac Cine measurement and depends on the scale of the pulsating vessel. Since the navigator signal depends on changes of blood signal within the slice, the trigger quality is sensitive to the applied flip angle. Robust trigger signals were only found with flip angles of 30° and higher. With flip angles less then 20°, the retrospective triggering often failed.

#### Temporal resolution

The monitoring of the pulse-wave propagation requires high temporal resolution. With the triggered interleaved PC-Cine measurements described in [4] a frame rate of 1000 frames/s can be achieved. Since always a fixed number of 100 time frames were reconstructed, the frame rate of the retrospective measurement depends on the heart rate. In the examined group the average cardiac interval was 101-115 ms, therefore the absolute frame rate was 870-990 frames/s, which was sufficient to take 5-8 data points during the upstroke of the systolic flow for the linear fit. For measurements of animals with lower heart rates (175 - 200 ms) one should increase the number of reconstructed Cine frames to ensure sufficient temporal resolution, which might result into longer measurement times.

**Figure 9:**
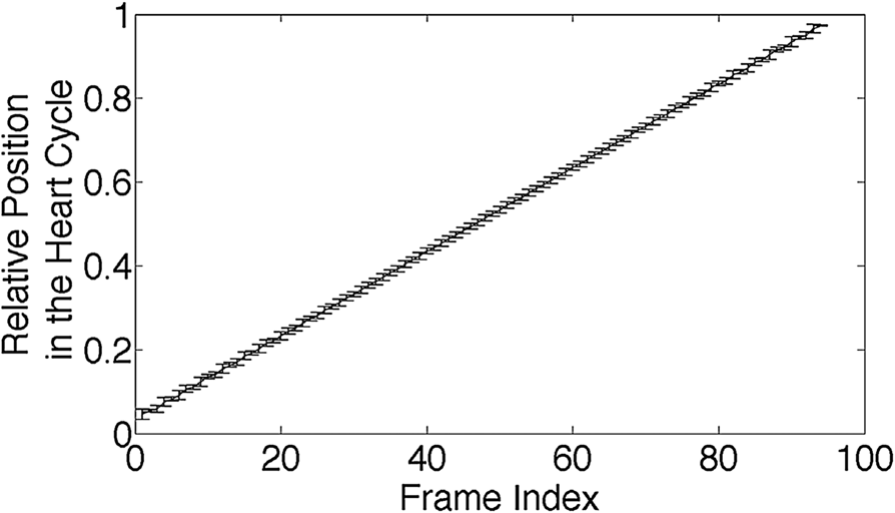
**Temporal blurring of the reconstructed relative time frames.** Average position in the heart cycle with standard deviation as a function of the reconstructed relative time frames. Since the mean variation is 0.75% of the heart cycle, the Cine frames have an overlap of 50%.

The native temporal resolution of the retrospective measurement is restricted to the repetition time of the read-out, which was 3 ms in this experiment. However, since the heart rate shows small variations due to naturalfluctuations of the heart function, the sampled states in the cardiac cycle slightly vary over the time, allowing a denser sampling of the aortic motion. As a result the radial projections could be assigned to relative positions in the heart cycle using a sliding window width of only 1 ms. Furthermore one could implement small TR variations in the timing of the pulse sequence to randomize even more the sampling of the heart periods. In Figure 9 the average positions in the heart cycle and the standard deviations for the heart cycle positions of the selected radial projections of one retrospective Cine measurement are plotted as a function of the frame index (between 1 und 100). The mean variation per frame was ±0.75% of the heart cycle, which corresponds to a deviation of ≈±0.8 ms. Since the temporal frames therefore had an overlap of ≈50%, the reconstructed Cine is slightly blurred and the measured area-and flow-curves appear to be smoothed. However, a low-pass filter is usually applied to the flow data of the triggered measurements as well to suppress high frequency random noise [4], which results into a similar smoothing effect.

#### Measurement time

A drawback of the triggered Cartesian PWV measurement is the long scan time. To achieve a temporal resolution of 1 ms, 5 segmented Cines were necessary. The average scan time of the Cartesian experiment was 6.7±1.1 minutes (mean ± SE). Since the early systolic time frame is very short, only few data points can be used for linear fitting. Therefore the heart rate has to be kept constant during the whole measurement. With the use of a highly asymmetric radial trajectory, the scan time of the triggered PWV measurement could be reduced to 4.3±0.6 minutes without losing temporal and spatial resolution. Using the retrospective measurement the whole PWV experiment can be done in only 2.9 minutes, which is more than two times faster than the triggered Cartesian protocol.

#### Contrast

Compared to the prospectively triggered methods, where the MRI signal does not reach the steady state due to cardiac and respiratory gating, the MRI signal of the retrospective flow measurement is in steady state all the time. Therefore each retrospectively reconstructed image contains a comparable contrast, while contrast changes are noticeable with the triggered and respiratory-gated measurements. In addition, since the surrounding static tissue appears to be darker than with the triggered sequences, the retrospectively triggered method enables an easier segmentation of the aorta.

#### Deviations of the radial trajectory

**Figure 10:**
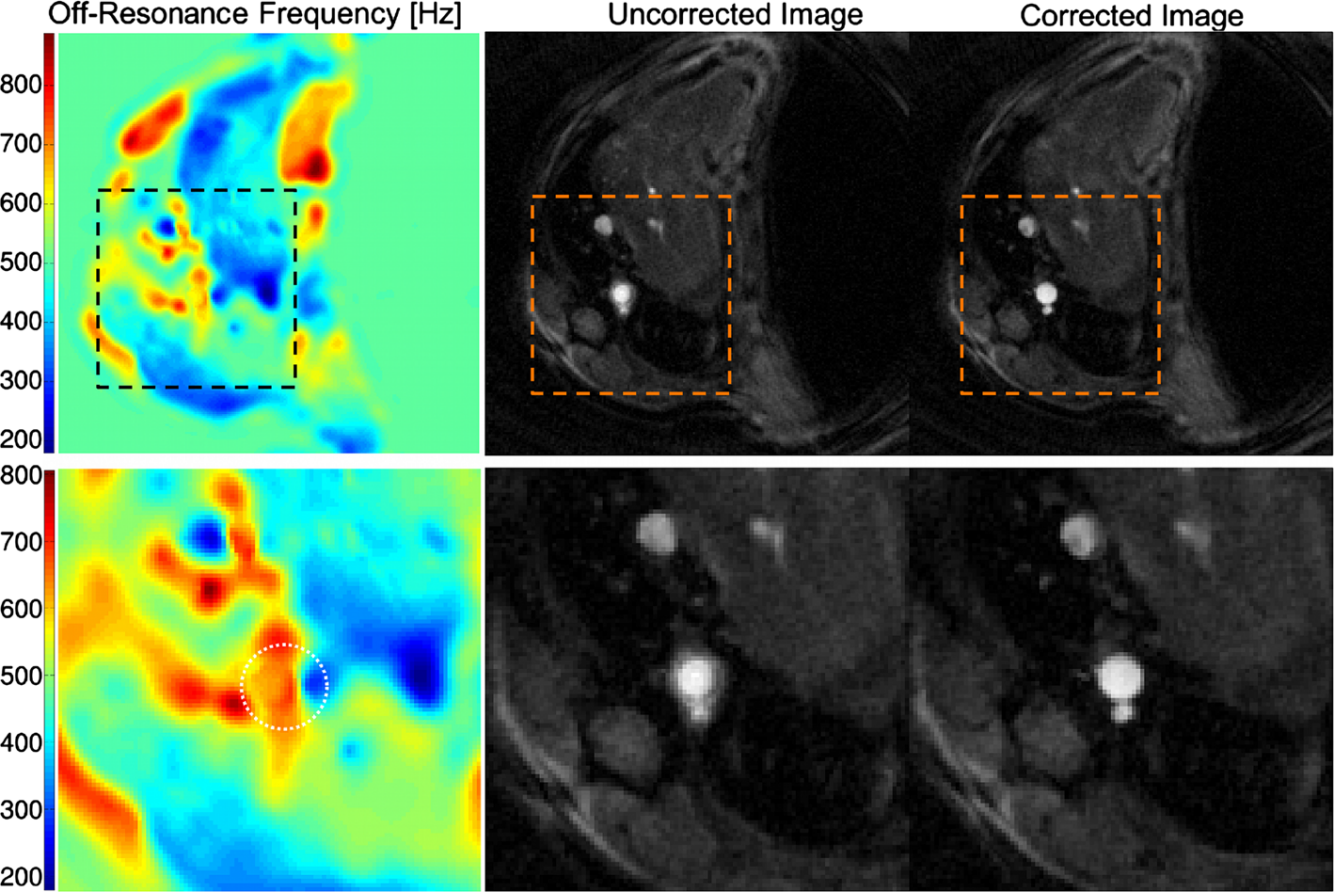
**Off-resonance correction reconstruction, which was determined with two gradient echo images with echo time difference *Δ*TE=0.5 ms.** Above: complete map. Below: section around the aorta. Middle: Uncorrected magnitude image of the radial measurement. Around the aorta severe blurring artifacts are visible. Right: Using the measured off-resonance map and the previously mentioned algorithm the radial images can be de-blurred.

The retrospective technique does not require external ECG probes and is therefore less sensitive to animal handling. However, off-resonance and local field inhomogeneities can compromise the quality of the reconstructed images and lead to deviations of the radial trajectory. Using highly asymmetric projections, the echo time can be shortened from 1.7 ms to 1.2 ms, reducing the effect of inhomogeneities and flow on the measurement. With the aid of B_0_ maps remaining susceptibility artifacts can be corrected retrospectively. To demonstrate the effect of the B_0_ correction an exemplary map of the off-resonance frequencies $\mathrm{\Delta f}(\vec{r})=\frac{\Delta \omega_{0}(\vec{r})}{2\pi }$ is shown in Figure 10 (top: complete map, bottom: section around the aorta). Around the aorta the frequency offset lies around 600 Hz, causing severe blurring artifacts in the uncorrected radial image which make an segmentation of the vessel impossible (see images in the middle). Using the measured B_0_ map and the algorithm mentioned above the blurring can be significantly reduced, allowing a better distinction of the vessel from the surrounding tissue (see right images). However, the acquisition of the required triggered gradient-echo images for this optional B_0_ correction results into an additional measurement time of 1-2 minutes. Even with correction, B_0_ inhomogeneities remain the main cause for uncertainties of aorta segmentation and difficulties with PWV measurements in vessels close to lung tissue. However, issues with susceptibility artifacts in vessels nearby lung tissue were also reported for the triggered Cartesian measurement described in [4]. Another cause of trajectory deviations are timing errors caused by delays of the spatial encoding gradients [11]. By measuring the gradient switching times trajectory distortions can be reduced, however, residual trajectory errors and errors of the phase contrast maps caused by eddy currents [12,27] can remain.

#### Advantages of the radial acquisition

In [28] and [29] a Cartesian self-gating method was proposed which measures a navigator echo during refocusing of the slice-selection gradient without the need of non-uniform trajectories. In [8] the rectilinear self-gating signal could be demonstrated to be more robust than the radial signal since it does not suffer from trajectory off-center effects. However, in this method the acquisition of the navigator signal was achieved by a temporal separation of the slice-refocusing gradient and the read dephase and phase encoding gradients. With a flow-encoding sequence based on a tripolar slice-selection gradient and a bipolar flow-encoding gradient this separation would result in an undesirable prolonging of the read-out duration and in a longer echo time. With the radial self-gating sequence proposed in this work a self-gating signal can be acquired without extending the timings of the flow-encoding sequence. Through the application of high echo asymmetry and short read dephase gradients the echo time could be reduced, which helps to reduce misregistration effects due to blood flow [30]. In addition, since every radial projection samples the k-space center and therefore has equal information content, an easier reconstruction of time frames is possible compared to the retrospective Cartesian sampling. As a further advantage, the proposed self-gated flow-encoding sequence profits from the advantages of the radial trajectory, due to the higher incoherency of the flow and motion artifacts [31,32] and a higher robustness against undersampling [33].

## Conclusions

We present a robust self-gated PC-Cine method that enables a measurement of the local pulse-wave velocity in the murine aorta without the need of additional triggering hardware. This new technique allows the reconstruction of high-resolution Cines of the aortic motion with a frame rate comparable to triggered methods achieving similar results for the PWV values.

## Abbreviations

PC: Phase-contrast; PWV: Pulse-wave velocity; VENC: Maximum Encoding Velocity; WT: Wild-type; CSA: Cross-sectional area.

## Competing interests

The authors declare that they have no competing interests.

## Authors’ contributions

PW developed the radial flow-encoding sequences and conducted all experiments. ER, WRB, VH and PMJ enrolled and organized this study. TK and VH helped with the data evaluation. XH and VH helped with the pulse-sequence development, XH, CBM and FTG provided the software for trigger signal evaluation. All authors took part in critical review and drafting of the manuscript and have read and approved the final manuscript.

## Supplementary Material

Additional file 1 **A Powerpoint animation to illustrate how the golden-ratio projections are sorted into golden-angle-distributed sublists.** In the animation only 4 sublists with 4 projections each are shown for an easier visualization. In a real retrospective flow measurement 16000 projections are sorted into 33-38 sublists with 420-475 projections each. Click here for file
