# Supplementary material for: Fast retrospectively triggered local pulse-wave velocity measurements in mice with CMR-microscopy using a radial trajectory
Source: J Cardiovasc Magn Reson. 2013 Oct 1;15(1):88. doi: 10.1186/1532-429X-15-88 (PMC3850985; doi:10.1186/1532-429X-15-88)
Supplement: Additional file 1 — A Powerpoint animation to illustrate how the golden-ratio projections are sorted into golden-angle-distributed sublists. In the animation only 4 sublists with 4 projections each are shown for an easier visualization. In a real retrospective flow measurement 16000 projections are sorted into 33-38 sublists with 420-475 projections each. [file 1532-429X-15-88-S1.PPTX]

## Slide 1
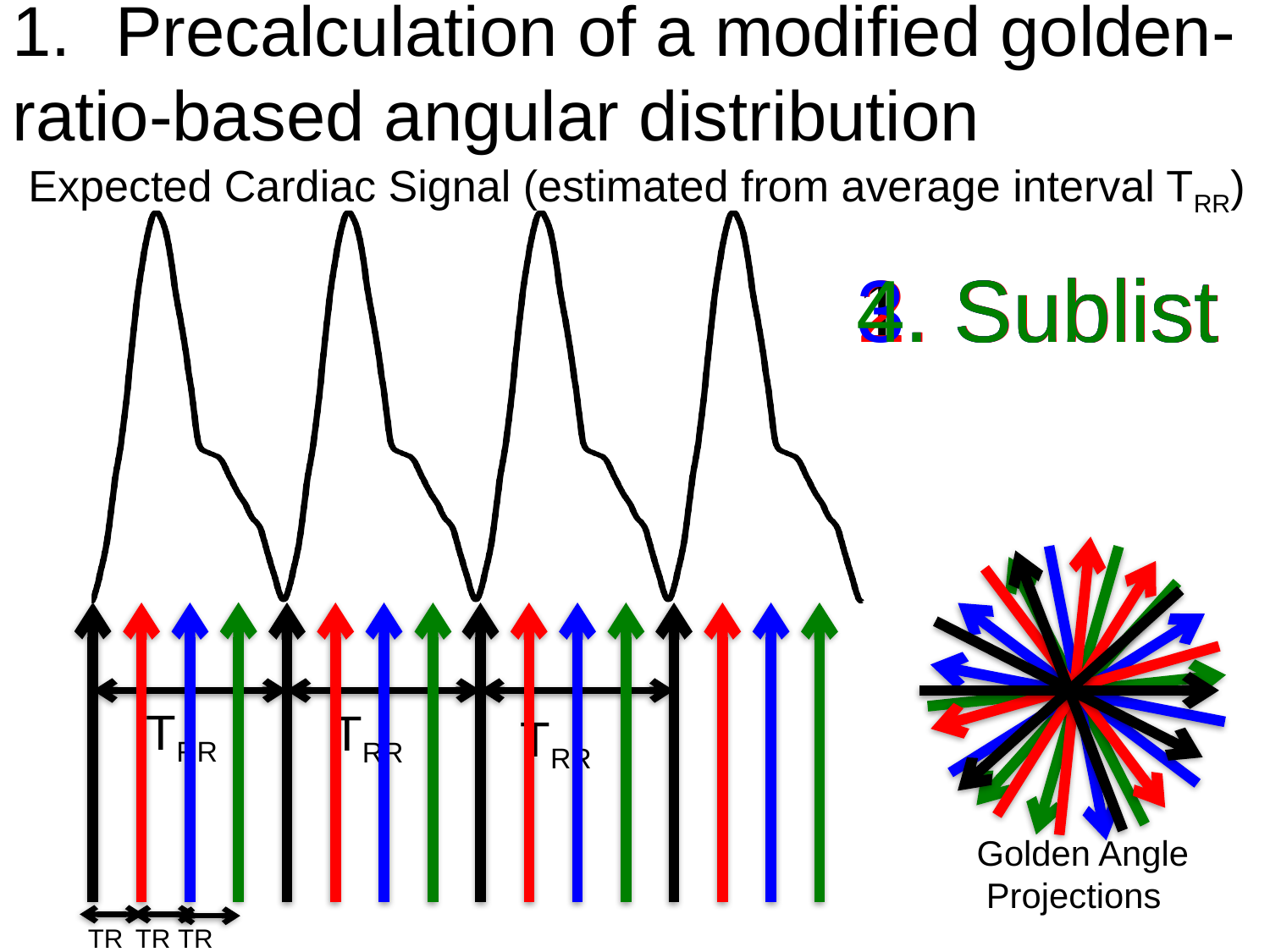

Precalculation of a modified golden-
ratio-based angular distribution
Expected Cardiac Signal (estimated from average interval TRR)
3. Sublist
4. Sublist
1. Sublist
2. Sublist
TRR
TRR
TRR
Golden Angle
 Projections
TR
TR
TR

## Slide 2
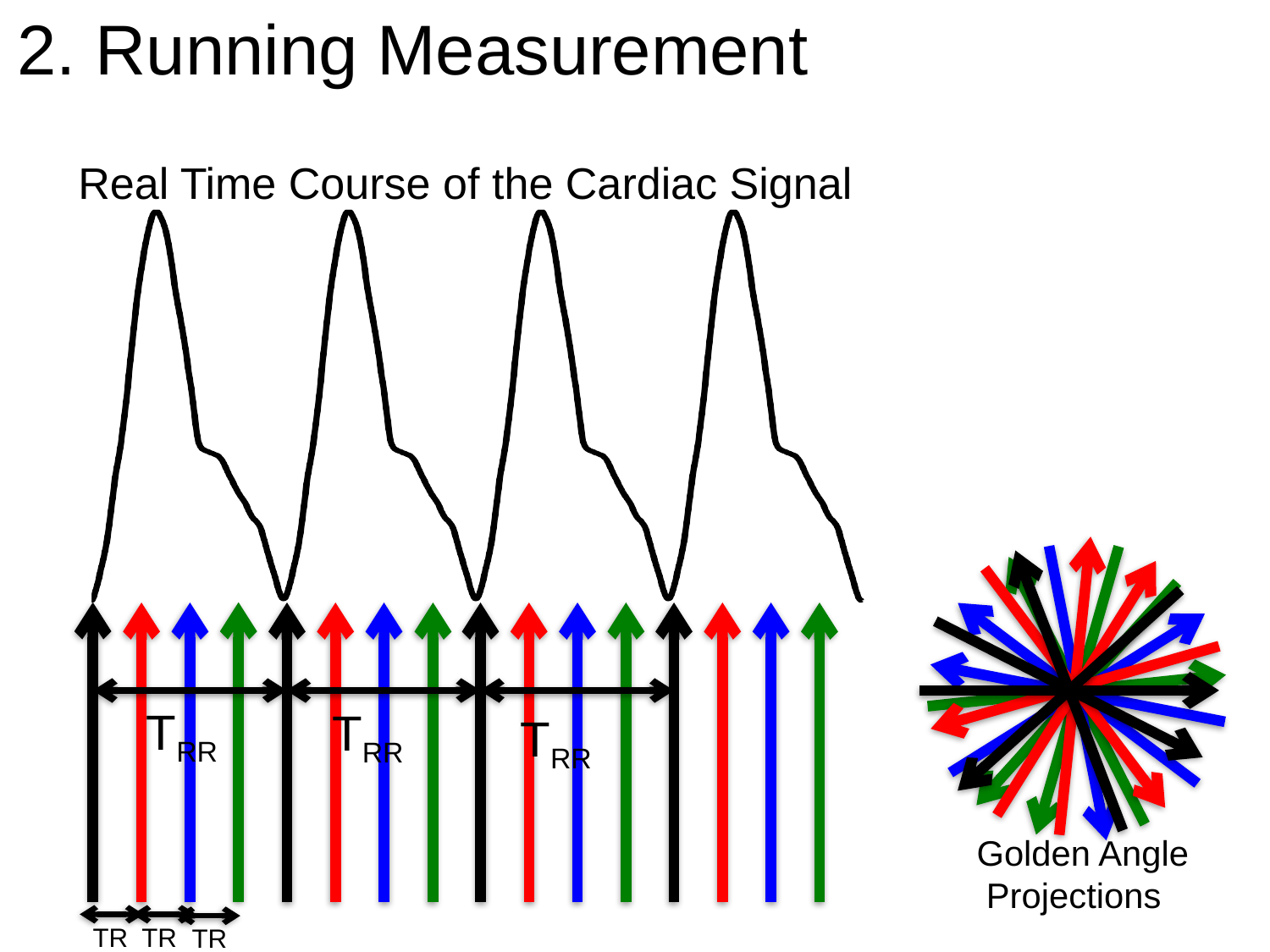

2. Running Measurement
Real Time Course of the Cardiac Signal
TRR
TRR
TRR
Golden Angle
 Projections
TR
TR
TR
